# Subjective Evaluation of Right Ventricular Systolic Function in Hypoplastic Left Heart Syndrome: How Accurate Is It?

**DOI:** 10.1016/j.echo.2012.09.020

**Published:** 2013-01

**Authors:** Hannah R. Bellsham-Revell, John M. Simpson, Owen I. Miller, Aaron J. Bell

**Affiliations:** a Department of Paediatric Cardiology, Level 6 Evelina Children's Hospital, London, United Kingdom; b Division of Imaging Sciences and Biomedical Engineering, Rayne Institute, Lambeth Wing, St. Thomas' Hospital, London, United Kingdom

**Keywords:** Magnetic resonance imaging, Echocardiography, Experience, Hypoplastic left heart syndrome, CI, Confidence interval, EF, Ejection fraction, HLHS, Hypoplastic left heart syndrome, MRI, Magnetic resonance imaging, RV, Right ventricular

## Abstract

**Background:**

The geometry and heterogeneity of the right ventricle in hypoplastic left heart syndrome makes objective echocardiographic assessment of systolic function challenging. Consequently, subjective echocardiographic assessment of right ventricular (RV) function is still routinely undertaken. The aims of this study were to compare this with magnetic resonance imaging (MRI), investigate the impact of experience and training on the accuracy of subjective assessment, and critically analyze the role of echocardiography to detect impaired systolic function.

**Methods:**

A retrospective analysis of prospectively acquired data was performed. Children with hypoplastic left heart syndrome underwent routine preoperative cardiac MRI and echocardiography under the same general anesthetic. Echocardiograms were reviewed, and members of the congenital heart disease team with differing echocardiography experience subjectively graded RV systolic function (good, moderate, or poor). This was compared with MRI-derived ejection fraction.

**Results:**

Twenty-eight patients at different palliative stages were included. Twenty-eight observers were divided into five experience categories (congenital heart disease junior trainees to attending cardiologists). Median agreement was 47.6% (range, 31.4%–58.2%), with the lowest agreement among junior trainees and the highest among attending cardiologists. When used as a screening test for poor RV systolic function, the median sensitivity of echocardiography was 0.89 (range, 0.86–0.96), and median specificity was 0.45 (range, 0.26–0.55). The highest sensitivity was observed among junior trainees but with the lowest specificity. The highest specificity was observed among attending cardiologists (0.55).

**Conclusions:**

Agreement between echocardiographic and MRI RV ejection fraction improves with experience but remains suboptimal. When used as a screening test for poor RV function, echocardiography is sensitive, but specificity is heavily influenced by operator experience.

Hypoplastic left heart syndrome (HLHS) describes a spectrum of underdevelopment of the left heart that renders it incapable of supporting the systemic arterial circulation.^1^ The current management approach includes three staged surgeries^2^ carried out over the first few years of the child's life; the resulting circulation is maintained by a systemic right ventricle. Impaired right ventricular (RV) systolic function is associated with poor outcomes,^3^ so the early detection of cardiac dysfunction has important implications for future management and prognosis. Assessment of RV function is an essential part of evaluation in both the acute inpatient setting and outpatient review. The geometry and heterogeneity of the single right ventricle in patients with HLHS make reliable and reproducible echocardiographic assessment of systolic function challenging.^4^

The “gold standard” for the measurement of intrinsic RV contractility is by conductance catheterization, but this is not used in routine clinical practice. In the clinical care of patients with HLHS, cardiac magnetic resonance imaging (MRI)^5^ is the imaging method used to provide objective measurement of ventricular volumes, ejection fraction (EF), and flow. MRI usually requires general anesthesia or sedation in younger children and is expensive, is not portable, and requires a level of expertise not universally available. Despite these universal limitations of MRI, it is used routinely at our institution^6^ to assess this patient group before hemi-Fontan and Fontan surgery, or if there are concerns regarding anatomic or functional complications.

Although several echocardiographic indices have been investigated for the assessment of RV function,^5^ there are still significant limitations,^7^ and in routine clinical practice, evaluation of RV function is still based predominantly on subjective assessment. Previous studies have attempted to describe established RV functional assessment parameters in patients with HLHS, but small numbers and multiple confounders (operative stage, left ventricular morphology) have meant that these are not necessarily widely applicable.^3,8-11^ A previous study of subjective assessment of single ventricular function (including single left, single right, and indeterminate ventricles) showed poor correlation between qualitative assessment of function and MRI, with subjective assessment concordant in fewer than half of cases.^12^

The first aim of this study was to investigate the accuracy of subjective echocardiographic assessment of systolic RV function in patients with HLHS, when compared with cardiac MRI–derived EF (Videos 1–2 [available at www.onlinejase.com]). As secondary aims, we sought to investigate the impact of observer training and experience on the correlation of echocardiographic and MRI methods, as well as the performance of echocardiography when used as a screening test to detect reduced RV EF measured on MRI.

## Methods

Ethical and institutional approval was granted and informed consent was obtained from parents or legal guardians. All patients with HLHS (defined as mitral stenosis or atresia with aortic stenosis or atresia and atrioventricular and ventriculoarterial concordance) undergoing cardiac MRI under general anesthesia were included in the study. At our institution, cardiac MRI is routinely used in combination with echocardiography before hemi-Fontan and total cavopulmonary connection as well as for patients after total cavopulmonary connection. Therefore, all patients with HLHS undergo MRI before hemi-Fontan and total cavopulmonary connection. Patients were excluded if parental consent was not given or if there was cardiovascular instability during the MRI scan, precluding additional time for echocardiography. Data were prospectively acquired but retrospectively analyzed for this study.

### MRI Acquisition

MRI scans were performed on a Philips 1.5-T Achieva scanner (Philips Medical Systems, Best, The Netherlands) and were reevaluated using ViewForum EWS version 2.0 (Philips Medical Systems, Best, The Netherlands). Two-dimensional steady-state free precession cine imaging oriented to the short axis of the right ventricle was used to calculate ventricular volumes using the disk summation method. End-diastolic and end-systolic contours were manually traced (excluding major trabeculations from the volume) to determine end-diastolic volume, end-systolic volume, stroke volume, and EF.^13^ MRI EF was categorized as used in our clinical department: good function (≥50%), moderate function (40%–49%), or poor function (<40%).

### Echocardiographic Acquisition

Echocardiography was undertaken immediately after the MRI scan under the same general anesthetic to avoid potential physiologic changes. Comprehensive echocardiography was performed on a Philips iE33 ultrasound system (Philips Medical Systems, Andover, MA). Subcostal, apical, long-axis, and short-axis views were obtained, whenever acoustic windows permitted. Two-dimensional cine images (no color) were anonymized and reviewed by a range of observers working within the department of congenital heart disease with varying levels of experience of echocardiography in this patient group. Observers were blinded to the MRI results. Each observer was asked on the basis of the images to categorize global RV systolic function as good, moderate, or poor consistent with the normal subjective description in clinical use at our institution. This method has been used previously to compare echocardiography and MRI in both the biventricular and single-ventricle setting.^12,14^

### Observer Grouping

Observers were grouped by experience level: residents with <6 months' exposure to echocardiography (*n* = 5), junior fellows with <3 years of training (*n* = 6), senior fellows with >3 years of training (*n* = 5), cardiac physiologists (*n* = 5), and attending cardiologists (*n* = 7). Further delineation of the experience of each group is given in the Appendix.

### Statistical Analysis

Observers were scored on the basis of the concordance of their visual assessment of RV systolic function on echocardiography with MRI EF (0 if concordant, 1 if a single functional echocardiographic grade different, and 2 if functional echocardiographic grade differed by two grades compared with the MRI scan). For example, if an MRI EF was good (>50%) and the observer rated it as poor echocardiographically, this was considered two grades different. Each observer was given a total score (calculated from the total of grades different); this was then averaged for each observer experience group. Thus, a low score means that there was a high level of agreement between the echocardiographic assessment and MRI, and a high score indicates worse agreement between the echocardiographic and MRI methods. To evaluate variability in assessment in each observer group, intraclass correlation coefficients were calculated within in each observer group (two-way random, absolute agreement).

An important clinical consideration is the ability of echocardiography to accurately detect reduced RV systolic function. If an RV EF of <50% assessed by MRI is regarded as “reduced function,” sensitivity, specificity, positive predictive value, and negative predictive value of subjective echocardiographic function were calculated for each observer group. Echocardiographically assessed moderate or poor function was taken to indicate reduced function.

All MRI scans were analyzed by two observers (H.R.B.-R. and A.J.B.). All echocardiograms were graded for quality by the same two observers. Scans were graded from 1 (poor quality) to 4 (excellent quality). Intraclass correlation coefficients were calculated to assess interobserver variation for MRI analysis (two-way random, absolute agreement).

## Results

Twenty-eight patients with HLHS underwent echocardiography under the same general anesthetic as MRI between July 2007 and January 2009. All patients had magnetic resonance and echocardiographic images available for analysis. The demographics and MRI results are shown in Table 1. Twenty-three patients (82%) had MRI EFs ≥ 50%, four (14%) had MRI EFs of 40% to 49%, and one (4%) had an MRI EF < 40%. The median MRI EF for the whole group was 59% (range, 33%–78%).

Interobserver variability for MRI analysis showed good concordance. Intraclass correlation coefficients were 0.945 (95th confidence interval [CI], 0.741–0.91), 0.952 (95% CI, 0.779–0.984), 0.926 (95% CI, 0.780–0.970), and 0.885 (95% CI, 0.764–0.946) for end-diastolic volume, end-systolic volume, stroke volume, and EF, respectively.

**Figure 1:**
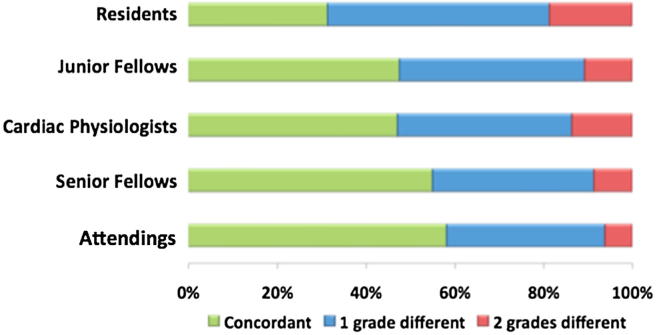
Increased correlation between MRI-derived EF and subjective echocardiographic assessment of RV function with increasing operator experience. Concordant = visual assessment grade concordant with MRI EF grade; one grade different = visual assessment grade one grade higher or lower than MRI EF grade; two grades different = visual assessment grade two grades higher or lower than MRI EF grade.

When evaluating the echocardiograms, there was a trend toward improved concordance with increasing experience (Figure 1). Attending cardiologists assessed the grade of function the same as MRI in 58.2% of patients, compared with 55% of senior fellows, 47.1% of cardiac physiologists, 47.6% of junior fellows, and 31.4% of residents. Concordance within each observer group also increased with increasing experience, from 0.674 (95% CI, 0.440–0.831) in residents to 0.876 (95% CI, 0.789–0.936) in attending cardiologists. Twenty-one echocardiograms (75%) were rated as having good to excellent quality clips; all loops were included to reflect the normal clinical situation of varying quality of images. It was noted that concordance with MRI in those with less than good to excellent images was lower than those with good to excellent clips (Table 2).

For the analysis of the performance of subjective echocardiographic assessment to detect reduced RV EF (<50%) by MRI, the sensitivity, specificity, positive predictive value, and negative predictive value (on the basis of group averages) are shown in Table 3. All observer groups had sensitivity > 0.80, with the highest sensitivity (0.96) observed in residents. The specificity of the technique was highest for attending cardiologists (0.55) and lowest for residents (0.26).

## Discussion

In routine clinical practice in the outpatient clinic, inpatient ward, or perioperative setting, echocardiography is the mainstay of assessment of RV systolic function in patients with HLHS. Although many qualitative techniques to determine systolic and diastolic function have been described,^5^ these have not been fully investigated in patients with HLHS and are not used in routine clinical practice. Additionally, the echocardiographer must deal with limited views, particularly in the postoperative period, and with the compliance of infants and young children. Therefore, more simple indices such as tricuspid annular displacement^15^ and an operator-dependent subjective assessment of RV systolic function are still commonly used despite evidence to suggest that subjective evaluation of the ventricular function in single-ventricle circulations correlates poorly with MRI.^12^ Subjective assessment may also be affected by differences seen in regional wall motion, as can be seen in those with different residual left ventricular morphologies.^16^

In this retrospective study, we sought to evaluate the accuracy of subjective assessment of RV systolic function in patients with HLHS, as well as investigating whether this was determined by operator experience. We have shown that visual assessment of RV systolic function has only a limited correlation with MRI EF but that this appears to improve with increasing levels of experience (Figure 1). Even in the most experienced group of echocardiographers, the echocardiographic grade of function was discordant with MRI assessment in >40% of cases. This variation with experience has obvious clinical implications, especially in the situation in which it may be a more junior member of the medical team performing the echocardiographic study. It highlights the importance of recognizing the skills and limitations of those undertaking echocardiography in this patient group and the need to ensure appropriate review.

An important role of echocardiography in the sequential assessment of patients with HLHS is to detect RV dysfunction. Our analysis of echocardiography to detect reduced RV EF (<50%) on the basis of MRI was undertaken to address this question. The observer group with the highest sensitivity to identify reduced RV EF was the residents, who have the least echocardiographic experience. This finding was matched, however, by the lowest specificity for residents, in contrast to the highest specificity observed among attending cardiologists, the most experienced group. We speculate that because of the important clinical implications of RV dysfunction in this patient group, that less experienced observers will “overcall” echocardiographically reduced RV systolic function to avoid missing patients with clinically significant ventricular impairment. More experienced observers may be more likely to trust their assessments of echocardiograms and be more confident that ventricular impairment is absent. If echocardiographic judgment of impaired ventricular function leads to further corroborative imaging such as MRI, then fewer patients would be referred for such imaging in the hands of more experienced observers.

The improvement seen with increasing experience is likely to be due to increased exposure to both echocardiography and MRI, often in the context of cardiac surgical conferences, where decisions are made with respect to patient management. Our results emphasize the need for more robust and objective echocardiographic tools for the sequential assessment of patients with HLHS. This is particularly important because of the noninvasive nature and ready repeatability of echocardiographic techniques for sequential assessment. Methods using Doppler tissue imaging and speckle tracking are currently being investigated^16-19^ and may prove useful in this group.

### Study Limitations

The majority of patients had MRI EF in the normal range. This is likely to represent a degree of selection bias, as those with very poor function are at greater risk for mortality.^3^ We included all patients at any surgical stage because of the small numbers of patients involved and to reflect clinical practice, but we acknowledge that there is a potential influence of different loading conditions. There are inherent problems in comparing a two-dimensional technique such as echocardiography, in which assessment of function is based on myocardial motion, with a three-dimensional technique using measurement of volume changes (MRI). RV EF on MRI does not necessarily reflect systolic function but has been used here as a surrogate in the absence of other proven reliable measures. We acknowledge additionally that we have compared a continuous variable (MRI EF using arbitrary cutoffs) with a categorical variable, but this method has been used in previous studies.^12,14^

## Conclusions

This study has shown that subjective assessment of RV function in patients with HLHS is limited but improves with increasing experience. This has important clinical implications, as junior staff members perform the majority of assessments, particularly in the immediate postoperative period, and emphasizes the importance of ongoing training and supervision. Objective echocardiographic measures to reliably measure RV function in these patients are required. Ideally, these would be simple, reproducible, and not altered by the patient's operative stage, loading conditions, or morphologic subtype.

## Figures and Tables

**Table 1:** Patient demographics and MRI results

Stage	*n* (%)	Age (mo)	Weight (kg)	Saturation (%)	EDVi (mL/m^2^)	ESVi (mL/m^2^)	SVi (mL/m^2^)	COi (L/min/m^2^)	EF (%)
Post-Norwood∗	14 (50%)	3 (2–5)	4.98 (3.42–6.83)	78 (72–98)	92 (48–113)	47 (16–57)	46 (26–60)	4.8 (2.2–6.7)	55 (33–63)
Post–hemi-Fontan	11 (39%)	27.5 (24–49)	12.85 (10.4–15.4)	84 (70–89)	79 (54–173)	32 (15–77)	51 (34–96)	4.3 (3.6–10)	62.5 (43–78)
Post-Fontan	3 (11%)	104 (76–111)	29 (23.6–32.5)	92 (92–95)	70 (52–75)	23 (20–29)	46 (29–50)	3.1 (2.7–4)	60 (60–70)

*COi*, Cardiac output indexed to body surface area; *EDVi*, end-diastolic volume indexed to body surface area; *ESVi*, end-systolic volume indexed to body surface area; *SVi*, stroke volume indexed to body surface area.

Data are expressed as median (range).

∗ All patients had undergone a classical Norwood procedure with a modified right Blalock-Taussig shunt.

**Table 2:** Clip quality and concordance

Clip quality	Number of clips	Total discordance	Average discordance
Moderate/poor	7	178	25.4
Excellent/good	21	312	14.9

Each clip was marked 0 for same as MRI, 1 for one grade different from MRI, and 2 for two grades different from MRI. Total discordance calculated from the sum of all observers for each clip.

**Table 3:** Specificity, sensitivity, PPV, and NPV

Group	Sensitivity	Specificity	PPV	NPV
Attending cardiologists	0.86	0.55	0.29	0.95
Senior fellows	0.80	0.54	0.27	0.93
Junior fellows	0.89	0.45	0.26	0.95
Cardiac physiologists	0.92	0.45	0.27	0.96
Residents	0.96	0.26	0.26	0.97

*NPV*, Negative predictive value; *PPV*, positive predictive value.

Based on the ability of subjective echocardiographic assessment to detect reduced function (function rated as moderate or poor) using MRI-derived EF <50% as the standard.

## Appendix

### Observer Experience Levels

Common to all groups, in any scan related to HLHS, a subjective assessment of RV systolic function as good, moderate, or poor is regularly incorporated into reports. All groups are also regularly present at clinical meetings at which both MRI and echocardiographic images are presented and reviewed.

### Residents

Residents include doctors within the pediatric cardiology department who do not perform echocardiography but are exposed to echocardiographic images and interpretation in the course of daily echocardiography meetings and observed studies. These are doctors who rotate through pediatric cardiology as part of their wider pediatric training.

### Junior and Senior Fellows

In the United Kingdom, certification in pediatric cardiology involves 5 years of training completely dedicated to pediatric cardiology. Echocardiography is a component of such training throughout all 5 years. In our department, most trainees are working toward or have completed the European accreditation in echocardiography in congenital heart disease. We have subdivided these trainees into those <3 years and those >3 years into their training.

### Cardiac Physiologists

Staff cardiac physiologists in the pediatric cardiology department all hold European accreditation in echocardiography of congenital heart disease and all have >5 years of clinical experience in this field. They work alongside the clinical team to provide further support for the clinical service. They undertake echocardiography in the outpatient and inpatient settings. They are broadly equivalent to cardiac sonographers in the United States.

### Attending Cardiologists

In the United Kingdom, attending pediatric cardiologists all undertake transthoracic echocardiography in both outpatient and inpatient settings. Subspecialized cardiologists take the lead in more advanced modalities (e.g., three-dimensional echocardiography). However, subjective estimation of RV function in HLHS is undertaken by all attending cardiologists.

## Supplementary Data

Video 1 Apical four-chamber view of a patient with HLHS showing good systolic RV function (MRI scan under the same general anesthetic showed an EF of >50%).

Video 2 Apical four-chamber view of a patient with HLHS showing poor systolic RV function (MRI scan under the same general anesthetic showed an EF of <40%).
