# Supplementary figures and images for: Subjective Evaluation of Right Ventricular Systolic Function in Hypoplastic Left Heart Syndrome: How Accurate Is It?
Source: J Am Soc Echocardiogr. 2013 Jan;26(1):52–6. doi: 10.1016/j.echo.2012.09.020 (PMC3548410; doi:10.1016/j.echo.2012.09.020)

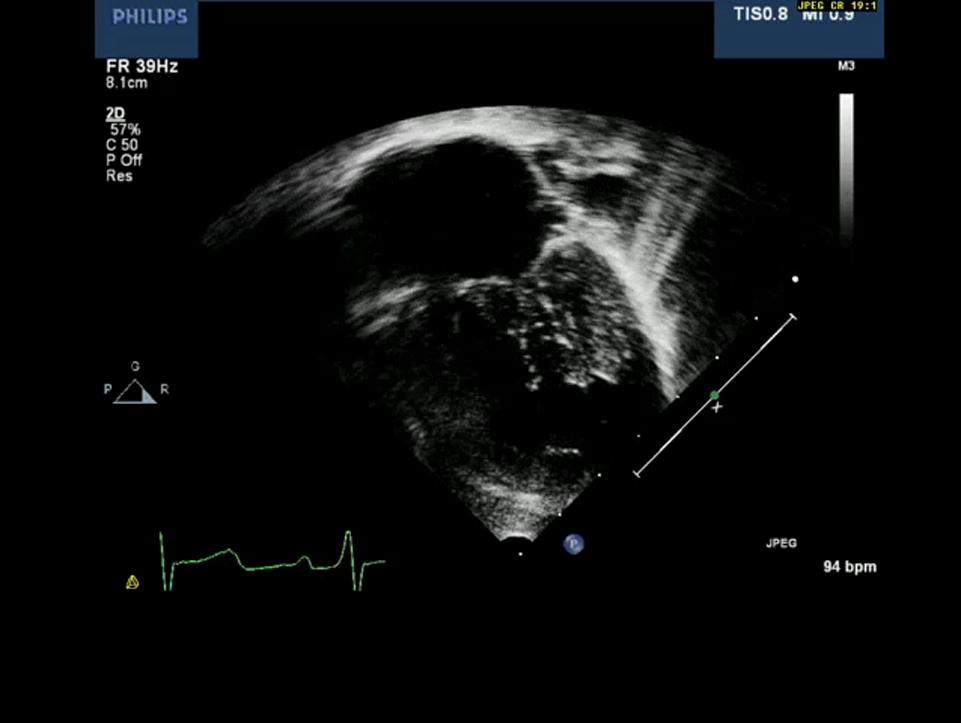

Supplement: Video 1 — Apical four-chamber view of a patient with HLHS showing good systolic RV function (MRI scan under the same general anesthetic showed an EF of >50%). [file mmc1.jpg]

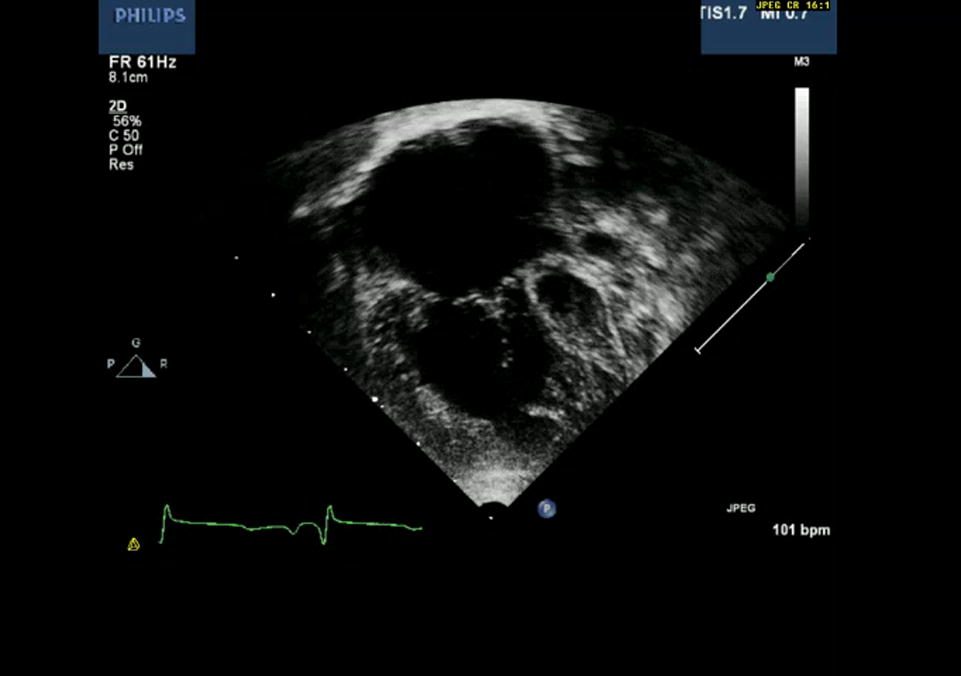

Supplement: Video 2 — Apical four-chamber view of a patient with HLHS showing poor systolic RV function (MRI scan under the same general anesthetic showed an EF of <40%). [file mmc2.jpg]
